# *Sm*10.3, a Member of the Micro-Exon Gene 4 (MEG-4) Family, Induces Erythrocyte Agglutination *In Vitro* and Partially Protects Vaccinated Mice against *Schistosoma mansoni* Infection

**DOI:** 10.1371/journal.pntd.0002750

**Published:** 2014-03-20

**Authors:** Vicente P. Martins, Suellen B. Morais, Carina S. Pinheiro, Natan R. G. Assis, Barbara C. P. Figueiredo, Natasha D. Ricci, Juliana Alves-Silva, Marcelo V. Caliari, Sergio C. Oliveira

**Affiliations:** 1 Departamento de Bioquímica e Imunologia do Instituto de Ciências Biológicas, Universidade Federal de Minas Gerais, Belo Horizonte, Minas Gerais, Brazil; 2 Instituto Nacional de Ciência e Tecnologia em Doenças Tropicais (INCT-DT), CNPq MCT, Salvador, Bahia, Brazil; 3 Departamento de Biologia Geral do Instituto de Ciências Biológicas, Universidade Federal de Minas Gerais, Belo Horizonte, Minas Gerais, Brazil; 4 Departamento de Biologia Celular do Instituto de Ciências Biológicas, Universidade de Brasília, Brasília, Distrito Federal, Brazil; 5 Departamento de Biointeração do Instituto de Ciências da Saúde, Universidade Federal da Bahia, Salvador, Bahia, Brazil; 6 Departamento de Patologia Geral do Instituto de Ciências Biológicas, Universidade Federal de Minas Gerais, Belo Horizonte, Minas Gerais, Brazil; McGill University, Canada

## Abstract

**Background:**

The parasitic flatworm *Schistosoma mansoni* is a blood fluke that causes schistosomiasis. Current schistosomiasis control strategies are mainly based on chemotherapy, but many researchers believe that the best long-term strategy to control disease is a combination of drug treatment and immunization with an anti-schistosome vaccine. Numerous antigens that are expressed at the interface between the parasite and the mammalian host have been assessed. Among the most promising molecules are the proteins present in the tegument and digestive tract of the parasite.

**Methodology/Principal Findings:**

In this study, we evaluated the potential of *Sm*10.3, a member of the micro-exon gene 4 (MEG-4) family, for use as part of a recombinant vaccine. We confirmed by real-time PCR that *Sm10.3* was expressed at all stages of the parasite life cycle. The localization of *Sm*10.3 on the surface and lumen of the esophageal and intestinal tract in adult worms and lung-stage schistosomula was confirmed by confocal microscopy. We also show preliminary evidence that r*Sm*10.3 induces erythrocyte agglutination *in vitro*. Immunization of mice with r*Sm*10.3 induced a mixed Th1/Th2-type response, as IFN-γ, TNF-α, and low levels of IL-5 were detected in the supernatant of cultured splenocytes. The protective effect conferred by vaccination with r*Sm*10.3 was demonstrated by 25.5–32% reduction in the worm burden, 32.9–43.6% reduction in the number of eggs per gram of hepatic tissue, a 23.8% reduction in the number of granulomas, an 11.8% reduction in the area of the granulomas and a 39.8% reduction in granuloma fibrosis.

**Conclusions/Significance:**

Our data suggest that *Sm*10.3 is a potential candidate for use in developing a multi-antigen vaccine to control schistosomiasis and provide the first evidence for a possible role for *Sm*10.3 in the blood feeding process.

## Author Summary

Schistosomiasis mainly occurs in developing countries and is the most important human helminth infection in terms of global mortality. This parasitic disease affects more than 200 million people worldwide and causes more than 250,000 deaths per year. Current schistosomiasis control strategies are mainly based on chemotherapy, but many researchers believe that the best long-term strategy for controlling schistosomiasis is a combination of drug treatment and immunization with an anti-schistosome vaccine. Consequently, significant effort has been dedicated to developing and characterizing an anti-schistosome vaccine. Over the last five years, considerable data have been generated regarding the genomics, transcriptomics and proteomics of *Schistosoma mansoni*. In the present study, we characterize the *Sm*10.3 protein and evaluate its potential to protect against *S. mansoni* infection in a murine model. We demonstrate that *Sm*10.3 is primarily expressed during the stages of the parasite life cycle that involve infection and disease development in the human host. *Sm*10.3 is located on the surface of the digestive epithelia of adult female worms, an important host/parasite interface. Moreover, the vaccination of mice with r*Sm*10.3 confers partial protection against *S. mansoni*. Taken together, our data suggest that *Sm*10.3 may be a useful component of a multi-antigen vaccine against schistosomiasis.

## Introduction

Schistosomiasis occurs primarily in developing countries and is the most important human helminth infection in terms of global mortality. This parasitic disease affects more than 200 million people worldwide, causing more than 250,000 deaths per year [1]. Furthermore, schistosomiasis is responsible for the loss of up to 4.5 million DALYs (disability adjusted life years) annually [2]. Current schistosomiasis control strategies are mainly based on chemotherapy but, despite decades of mass treatment, the number of infected people has not decreased considerably in endemic areas [3]–[5]. The extent of endemic areas, constant reinfection of individuals and poor sanitary conditions in developing countries make drug treatment alone inefficient [6]. It is thought that the best long-term strategy for controlling schistosomiasis is through immunization with an anti-schistosome vaccine combined with drug treatment [7]. A vaccine that induces even a partial reduction in worm burdens could considerably reduce pathology and limit parasite transmission [8].

Currently, the most promising schistosome vaccine candidates are proteins located on the surface of the worms [9], such as the tegument proteins TSP-2 [10] and *Sm*29 [11]. The tegument is a dynamic host-interactive surface involved in nutrition, immune evasion/modulation, excretion, osmoregulation, sensory reception, and signal transduction [12], [13]. Other surface-exposed proteins with high potential as vaccine targets are located in the digestive tract of lung-stage schistosomula and adult worms [14]–[18].

In this study, we evaluated the potential of Sm10.3, a member of the micro-exon gene 4 (MEG-4) family, to serve as a component of a recombinant subunit vaccine. The *Sm*10.3 antigen was first described in 1988 [19], but the *Sm10.3* and MEG family genes were not completely characterized until the recent publication of the *S. mansoni* genome [20]. There are multiple copies of some MEGs in the *S. mansoni* genome, arranged as tandem, symmetrically organized exons with lengths that are a multiples of three bases (from 6 and 36 base pairs) [20], [15]. It is thought that this arrangement may lead to protein variation through alternative splicing. Moreover, most of the MEGs are up-regulated during the stages in the parasite life cycle that involve establishment in the mammalian host [15].

In this study, we determined that *Sm*10.3 is located on the surface of the digestive tract of *S. mansoni* adult female worms and lung-stage schistosomula. We detected higher levels of *Sm10.3* mRNA in the schistosomula stage of the parasite life cycle. We also show preliminary evidence that *Sm*10.3 plays a role in erythrocyte agglutination. Furthermore, we report that vaccination with r*Sm*10.3 induces a mixed Th1/Th2-type immune response in mice, which correlates with a reduction in the worm burden and liver pathology.

## Methods

### Ethics statement

All animal experiments were conducted in accordance with the Brazilian Federal Law number 11.794, which regulates the scientific use of animals, and IACUC guidelines. All protocols were approved by the Committee for Ethics in Animal Experimentation (CETEA) at Universidade Federal de Minas Gerais UFMG under permit 179/2010.

### Mice and parasites

Female C57BL/6 mice aged 6–8 weeks were purchased from the Federal University of Minas Gerais (UFMG) animal facility. *S. mansoni* (LE strain) cercariae were maintained in *Biomphalaria glabrata* snails at CPqRR (Centro de Pesquisa René-Rachou-Fiocruz) and prepared by exposing infected snails to light for 1 h to induce shedding. Cercarial numbers and viability were determined prior to infection using a light microscope.

### *Sm*10.3 cloning and antigen preparation

The plasmid pJ414 containing the sequence for r*Sm*10.3 (pJ414::*Sm*10.3) was manufactured by DNA 2.0 (https://www.dna20.com) using DNA2.0 optimization algorithms for expression in *Escherichia coli*. This plasmid was transformed into *E. coli* Rosetta-gami (Merck KGaA, Darmstadt, Germany) competent cells. Transformants harboring the designed plasmid were screened on LB agar plates containing ampicillin (50 µg/ml) and cloranphenicol (34 µg/ml) and the selected transformant was designated as r*Sm*10.3-Rosetta. One liter of r*Sm*10.3-Rosetta was cultured in a three-liter erlenmeyer on a rotary shaker at 200 rpm at 37°C to an optical density at 600 nm of approximately 0.5–0.8 and gene expression was induced by using 1 mM isopropylthiogalactoside (IPTG). After 5 h of induction, the bacterial cells were harvested by centrifugation at 4,000× g for 20 min. Using gently vortexing or pipetting, the pellet was resuspended in 50 ml of 10 mM Na_2_HPO_4_, 10 mM NaH_2_PO_4_, 0.5 M NaCl and 20 mM imidazole. Subsequently, the cells were submitted to three cycles of sonication lasting 30 s each and centrifuged at 5400× g for 20 min. The r*Sm*10.3 was recovered solubilized in the supernatant and purified by affinity chromatography on a Ni-Sepharose column (Hitrap chelating 5 mL) using an AKTA explorer chromatography system (GE Healthcare, São Paulo, Brazil). After protein binding to the Ni-Sepharose column, washes with 50 mM imidazole were performed and the protein was eluted with 500 mM imidazole. Fractions containing the protein were determined through Bradford's method (Coomassie Protein Assay Kit, Pierce) and also SDS/PAGE-12% and dialyzed against PBS pH 7.0. The dialysis was carried out at 4°C using a Spectra/Por2 membrane (MWCO 6 to 8 kDa; Spectrum Medical Industries, Inc., Laguna Hills, CA). The recombinant protein was quantified using the Bradford's method and used as antigen for vaccination and immunological experiments. All reagents were purchased from Sigma-Aldrich, CO (St. Louis, MO, USA) unless otherwise specified.

### Real-time PCR

Total RNA was isolated from adult parasites, eggs, miracidia, cercariae or schistosomula using published procedures [21]. Total RNA was extracted with Trizol (Invitrogen, Carlsbad, CA, USA) according to the manufacturer's instructions. RNA samples were treated with DNAse, purified and concentrated using the RNeasy Micro Kit (QIAGEN, Valencia, CA, USA) according to the manufacturer's instructions. A 1.5 µg portion of each sample was reverse transcribed using the SuperScript*H* III First-Strand Synthesis SuperMix (Invitrogen, Carlsbad, CA, USA). Specific primer pairs (5′-CTT AAT CAA TAA GCC AAA GG-3′ and 5′-TAT TGA TTT GTC GTA ATA GT-3′) were designed using the Primer Express program (Applied Biosystems, Foster City, CA, USA) and default parameters and arbitrarily named primers 1 and 2, respectively. Real-time RT-PCR reactions were conducted in triplicate in a 20 µL volume containing 10 µL of Sybr Green PCR Master Mix (Applied Biosystems, Foster City, CA, USA), 160 nmol of each primer (primers 1 and 2) and 0.30 µL of the cDNA generated by reverse transcription. Real-time RT-PCR was performed using the 7300 Real-Time PCR System (Applied Biosystems, Foster City, CA, USA) and the following cycling parameters: 60°C for 10 min, 95°C for 10 min and 40 cycles of 95°C for 15 sec and 60°C for 1 min. A dissociation curve was generated using the following conditions: 95°C for 15 sec, 60°C for 1 min, 95°C for 15 sec and 60°C for 15 sec. Real-time data were normalized to the expression level of NADH dehydrogenase. *p*-values were determined by Student's *t*-test, using one-tailed distribution and heteroscedastic variance.

### SDS-PAGE and immunoblotting

Purified r*Sm*10.3 was analyzed on 15% polyacrilamide SDS-PAGE gels prepared and run as previously described [22]. The gel was then transferred to a nitrocellulose membrane [23]. The membrane was blocked with TBS-T (0.5 M NaCl−0.02 M Tris (pH 7.5), 0.05% Tween 20) containing 5% dry milk for 16 h at 4°C. The membrane was then incubated with a mouse monoclonal antibody to the 6×His-tag (GE Healthcare, Pittsburgh, PA, USA) diluted 1∶2,000 or a mouse polyclonal antibody to r*Sm*10.3 diluted 1∶1,000 for 1 h at room temperature. After three washes with TBS-T, the membrane was incubated in 1∶2,000 goat anti-mouse IgG conjugated to alkaline phosphatase (AP), then treated with a developing buffer containing nitroblue tetrazolium (NBT) and 5-bromo-4-chloro-3-indolyl-1-phosphate (BCIP). After the membrane was imaged, it was washed with distilled water and dried by sandwiching between two sheets of filter paper for storage. All reagents were purchased from Sigma-Aldrich, CO (St. Louis, MO, USA) unless otherwise specified.

### Immunization of mice

Six to eight week-old female C57BL/6 mice were divided into two groups of ten mice each. Mice were injected subcutaneously at the nape of the neck with 25 µg of r*Sm*10.3 protein or PBS (for the control group) on days 0, 15 and 30. The protein mixture was formulated using Complete Freund's Adjuvant (CFA) for the first immunization and Incomplete Freund's Adjuvant (IFA) for the last two immunizations (Sigma-Aldrich, CO, St. Louis, MO, USA).

### Immunolocalization of *Sm*10.3 in *S. mansoni* adult and worms and lung-stage schistosomula

For the microscopy studies, adult worms were recovered from perfused mice, and lung-stage schistosomula were prepared *in vitro* as described by Harrop & Wilson [24]. Parasites were fixed in Omnifix II (Ancon Genetics, St Petersburg, FL, USA) for sectioning. For the sectioning assays, 7 µm slices of Paraffin-embedded adult male or female parasites were deparaffinized using xylol and hydrated with an ethanol series, [25]. For experiments using *in vitro* cultured lung-stage schistosomula, a whole-mount protocol was chosen, lung stage schistosomula were treated with permeabilizing solution (0.1% Triton X-100, 1% BSA and 0.1% sodium azide in PBS pH 7.2) overnight at 4°C [25]. Following, permeabilized schistosomula and parasite sections were blocked with 1% BSA (bovine serum albumin) in PBST (phosphate buffered saline, pH 7.2 with 0.05% Tween-20) for 1 h and incubated with anti-r*Sm*10.3 serum diluted 1∶20 in blocking buffer. Serum from non-immunized mice was used as a negative control. The samples were washed three times with PBST and incubated with an anti-mouse IgG antibody conjugated to FITC (Molecular Probes, Carlsbad, CA, USA) diluted 1∶100 in blocking buffer containing rhodamine phalloidin (Molecular Probes, Carlsbad, CA, USA) to stain the actin microfilaments. The samples were washed four times and mounted with ProLong Gold anti-fading mounting medium containing DAPI (Molecular Probes, Carlsbad, CA, USA). Schistosomula were imaged on a Nikon A1R confocal microscope (60× NA1.4-CFI-Plan-Apo oil objective), and adult worms were imaged on a Nikon Eclipse Ti microscope from the Microscopy Center of the Biological Sciences Institute (CEMEL) at the Federal University of Minas Gerais (UFMG). All reagents were purchased from Sigma-Aldrich, CO (St. Louis, MO, USA) unless otherwise specified.

### Erythrocyte agglutination assays

Mice blood for the hemagglutination assays were collected and processed as described previously [26]. Blood was withdrawn from mice with a syringe, added to tubes containing EDTA (at a final concentration of 12 mM) and centrifuged for 15 min at 3000× g at room temperature. After centrifugation, the plasma was transferred to a fresh tube. In a second tube, erythrocytes were washed three times and suspended in PBS (pH 7.2) to a hematocrit of 20 or 40%. The erythrocyte suspensions were used for the agglutination assays on glass slides, in the hemagglutination endpoint dilution assays or in the hemagglutination kinetics assays.

Agglutination assays on glass slides was performed as previously described [26], the erythrocyte suspensions were prepared as above to a final hematocrit of 20% diluted in PBS. Next, 9 µl of each erythrocyte suspension was combined with 1 µl of r*Sm*10.3 containing 5 µg of purified protein, 1 µl of PBS or 1 µl of PBS containing 5 µg of r*Sm*29 (an unrelated *S. mansoni* antigen) as negative controls. Recombinant protein r*Sm*29 [11] was expressed with a 6×-histidine tag and purified in a similar way to r*Sm*10.3, as described in previous sections. The 10 µl mixture was loaded onto glass slides, covered with coverslips and immediately visualized using 10× and 40× objective lenses on a microscope equipped with a JVC TK-1270/RBG micro camera.

Hemagglutination endpoint dilution assays were performed as previously described [27], [28]. A serial two-fold dilution of 50 µl of r*Sm*10.3, r*Sm*29 and Concanavalin A solutions (protein concentrations ranged from 500 µg/mL to 0.97 µg/mL) in microtiter U-plates was mixed with 50 µl of a 2% suspension of mice erythrocytes in PBS at 37°C, resulting in a final erythrocytes suspension volume of 100 µl and a hematocrit of 1%. In the blank wells 50 µl of a 2% suspension of mice erythrocytes were mixed with 50 µl of PBS. Concanavalin A was used as positive control of the hemagglutination process [29]–[31] and r*Sm*29 (an unrelated *S. mansoni* antigen) as negative control. The results were read after approximately 1 h when the blank had fully sedimented. The endpoint was defined as the highest dilution showing complete hemagglutination. The hemagglutination titer, defined as the reciprocal of the highest dilution exhibiting hemagglutination, was defined as one hemagglutination unit. Specific activity is the number of hemagglutination units per mg of protein per milliliter [32].

Mice polyclonal antibodies raised against r*Sm*10.3 were used as inhibitor in hemagglutination inhibition assays according to a protocol previously described [33], with modifications. Twenty five microliters of r*Sm*10.3 solutions with three different protein concentrations (1000 µg/mL, 500 µg/mL and 250 µg/mL) were mixed with a serial two-fold dilution of 25 µl of anti- r*Sm*10.3 mice serum, ranging from 1∶1 to 1∶32 dilution and incubated at 37°C for 30 min. Following, 50 µl of a 2% suspension of mice erythrocytes in PBS was added to the wells and incubated for 1 h at 37°C.

The kinetics of the hemagglutination process were monitored by analyzing variations in turbidity [26]. Briefly, 100 µl of the erythrocyte suspension (in PBS) at a hematocrit of 5% was added to 96-well plates, and the agglutination was triggered by adding 0.5 to 5 µg of r*Sm*10.3 diluted in 100 µl of PBS, 100 µl of PBS containing 5 µg of Concanavalin A, as positive control of the hemagglutination process and 100 µl of PBS or 100 µl of PBS containing 5 µg of r*Sm*29 as negative controls, resulting in a final volume of 200 µl and a hematocrit of 2.5%. Plates were incubated at 37°C and spectrophotometric readings were taken at 655 nm every 13 s, with 8 s of shaking between each reading in a VersaMax Tunable Microplate reader (Molecular Devices, Sunnyvale, CA, USA). All reagents were purchased from Sigma-Aldrich, CO (St. Louis, MO, USA).

### Infection and worm recovery

Fifteen days after the final immunization, the mice were challenged with 100 cercariae (LE strain) by percutaneous exposure of the abdominal skin for 1 h. Forty-five days after the challenge, the adult worms were perfused from the portal veins, as described previously [34]. Two independent experiments were performed to determine protection levels. The degree of protection was calculated by comparing the number of worms recovered from each vaccinated group to the respective control group, using the following formula:
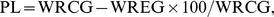
where PL indicates the protection level, WRCG indicates the number of worms recovered from the control group, and WREG indicates the number of worms recovered from the experimental group.

### Measurement of specific anti-r*Sm*10.3 antibodies

Following immunization, sera were collected from ten mice in each experimental group at two week intervals. The levels of specific anti-*Sm*10.3 antibodies were measured by indirect ELISA. Maxisorp 96-well microtiter plates (Nunc, Roskilde, Denmark) were coated with 5 µg/mL r*Sm*10.3 in carbonate-bicarbonate buffer (pH 9.6) for 16 hat 4°C, then blocked for 2 h at room temperature with 200 µL/well PBST (phosphate buffer saline, pH 7.2 with 0.05% Tween-20) plus 10% FBS (fetal bovine serum). One hundred microliters of serum diluted 1∶100 in PBST was added to each well, and the plates were then incubated for 1 h at room temperature. Plate-bound antibodies were detected using peroxidase-conjugated anti-mouse IgG, IgG1 and IgG2a (Southern Biotechnology, CA, USA) diluted 1∶10000, 1∶5000 and 1∶2000 in PBST, respectively. The color reaction was induced by adding 100 µL of 200 pmol OPD (o-phenylenediamine) in citrate buffer (pH 5.0) plus 0.04% H_2_O_2_ to each well for 10 min. The color reaction was stopped by adding 50 µL of 5% sulfuric acid to each well. The plates were read at 495 nm in an ELISA plate reader (BioRad, Hercules, CA, USA). All reagents were purchased from Sigma-Aldrich, CO (St. Louis, MO, USA) unless otherwise specified.

### Cytokine analysis

The cytokine experiments were performed using cultured splenocytes from individual mice immunized with r*Sm*10.3 or PBS (n = 4 for each group). The splenocytes were isolated from the macerated spleens of individual mice one week after the third immunization and washed twice with sterile PBS. After washing, the cells counts were adjusted to 1×10^6^ cells per well in RPMI 1640 medium (Gibco) supplemented with 10% FBS, 100 U/mL of penicillin G sodium and 100 µg/mL of streptomycin sulfate. The splenocytes were maintained in culture with medium alone or stimulated with r*Sm*10.3 protein (15 µg/mL), concanavalin A (ConA) (5 µg/mL), or LPS (1 µg/mL), as previously described [34]. The 96-well plates (Nunc, Roskilde, Denmark) were maintained in a 37°C incubator with a 5% CO_2_ atmosphere. The culture supernatants were collected after 24 h to measure IL-5 levels, after 48 h to measure TNF-α levels and after 72 h to measure IFN-γ and IL-10 levels. The cytokine measurement assays were performed using the DuoSet ELISA kit (R&D Systems, Minneapolis, MN) according to the manufacturer's instructions. All reagents were purchased from Sigma-Aldrich, CO (St. Louis, MO, USA) unless otherwise specified.

### Histopathological analysis

Following perfusion to recover the schistosomes, liver samples were collected from 8 animals each from the control and experimental groups to evaluate the effect of immunization on granuloma formation. The liver samples, which were taken from the central part of the left lateral lobe, were fixed with 10% buffered formaldehyde in PBS. Histological sections were performed using microtome at 6 µm and stained on a slide with picrosirius-haematoxylin-eosin (PSHE). The count of granulomas was performed at a microscope with 10× objective lens. Each liver section was scanned for calculating its whole area (mm^2^) using the ImageJ software (http://rsbweb.nih.gov/ij/index.html). For measurement of the total area of granulomas, a microscope with 10× objective lens was used; images were obtained through a JVC TK-1270/RBG microcamera attached to the microscope. Twenty granulomas with a single-well-defined egg were randomly selected, in each liver section and the granuloma area was measured using the ImageJ software. Granuloma fibrosis was analyzed using the software analysis getIT (Olympus Soft Imaging getIT) and images to illustrate the fibrosis area were edited using Adobe Photoshop software. All reagents were purchased from Sigma-Aldrich, CO (St. Louis, MO, USA).

### Statistical analysis

The results from the two experimental groups were compared by Student's *t*-test using the software package GraphPad Prism (La Jolla, CA). Bonferroni adjustments were included for multiple comparisons. The *p*-values obtained by this method were considered significant if they were <0.05.

### Accession numbers

*Sm*10.3 (M22346.1), *Sm*29 (AF029222.1), Tsp2 (AF521091.1).

## Results

### Expression profile of *Sm*10.3 at different stages in the *S. mansoni* life cycle

**Figure 1:**
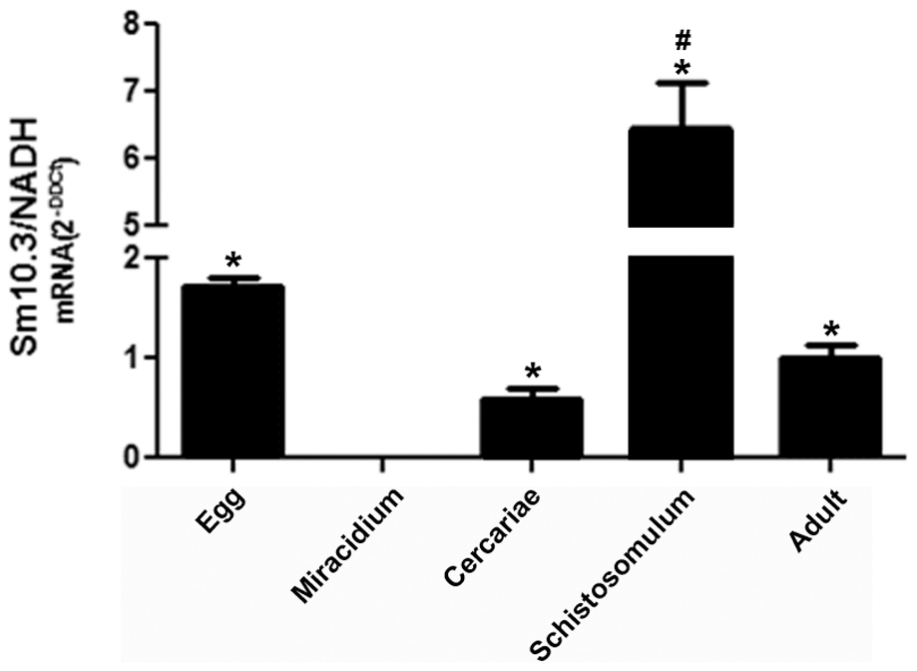
Expression profile of *Sm10.3* at different stages in the *S. mansoni* life cycle. Real time RT-PCR showing relative levels of *Sm10.3* transcripts at different stages in the *S. mansoni* life cycle (egg, miracidium, cercaria, schistosomulum and adult worm). Statistically significant differences compared to miracidia are denoted by asterisks, and statistically significant differences compared to eggs, cercariae and adult worms are indicated by # (*p*<0.05). Error bars indicate intra-assay standard deviation of means. Results are representative of two independent experiments.

The expression of the *Sm10.3* gene was detected by real-time PCR at different stages in the *S. mansoni* life cycle. The only stage during which *Sm*10.3 mRNA was not detected was the miracidium stage. The highest level of *Sm*10.3 mRNA expression was observed in lung-stage schistosomula. *Sm*10.3 expression was also detected in eggs, adult worms and cercariae, but at lower levels than in the schistosomula (Fig. 1).

### Expression and purification of r*Sm*10.3

**Figure 2:**
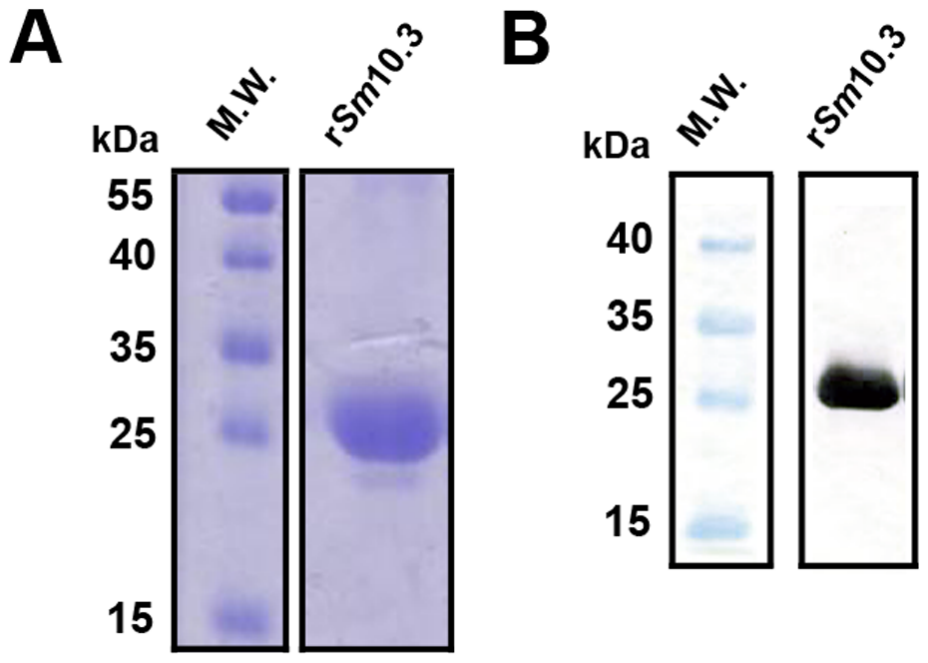
Heterologous expression and purification of r*Sm*10.3. (A) SDS-PAGE stained with Coomassie brilliant blue showing eluted and dialyzed r*Sm*10.3 after purification by Ni^2+^-charged column chromatography. Ten microliters were loaded per lane. The molecular weight protein standard (M.W.) is a broad range pre-stained ladder from BioRad. (B) Western blot analysis of r*Sm*10.3 proteins probed with monoclonal mouse anti-His tag antibodies. Ten micrograms of proteins were loaded per lane. The molecular weight protein standard (M.W.) is a broad range pre-stained ladder from BioRad.

Cloning and heterologous expression of the *Sm10.3* gene was performed as described in the material and methods section. Recombinant *Sm*10.3 (r*Sm*10.3) was purified from bacteria, and the first seven fractions that were eluted from an affinity chromatography column were combined and dialyzed in PBS pH 7.2 and then analyzed by SDS-PAGE followed by Coomassie blue staining (Fig. 2A). The strong band visible at approximately 26 kDa, the expected mass of the purified r*Sm*10.3, indicates the success of the purification protocol (Fig. 2A). To further evaluate the specificity of the purification procedure, the purified r*Sm*10.3 was analyzed by western blot using a mouse monoclonal anti-His tag antibody (Fig. 2B). The western blot analysis confirmed that the protein around 26 kDa had a histidine tag and this is the molecular weight expected for r*Sm*10.3 (Fig. 2A, B).

### *Sm*10.3 is localized on the surface of the intestinal tract of adult *S. mansoni*

**Figure 3:**
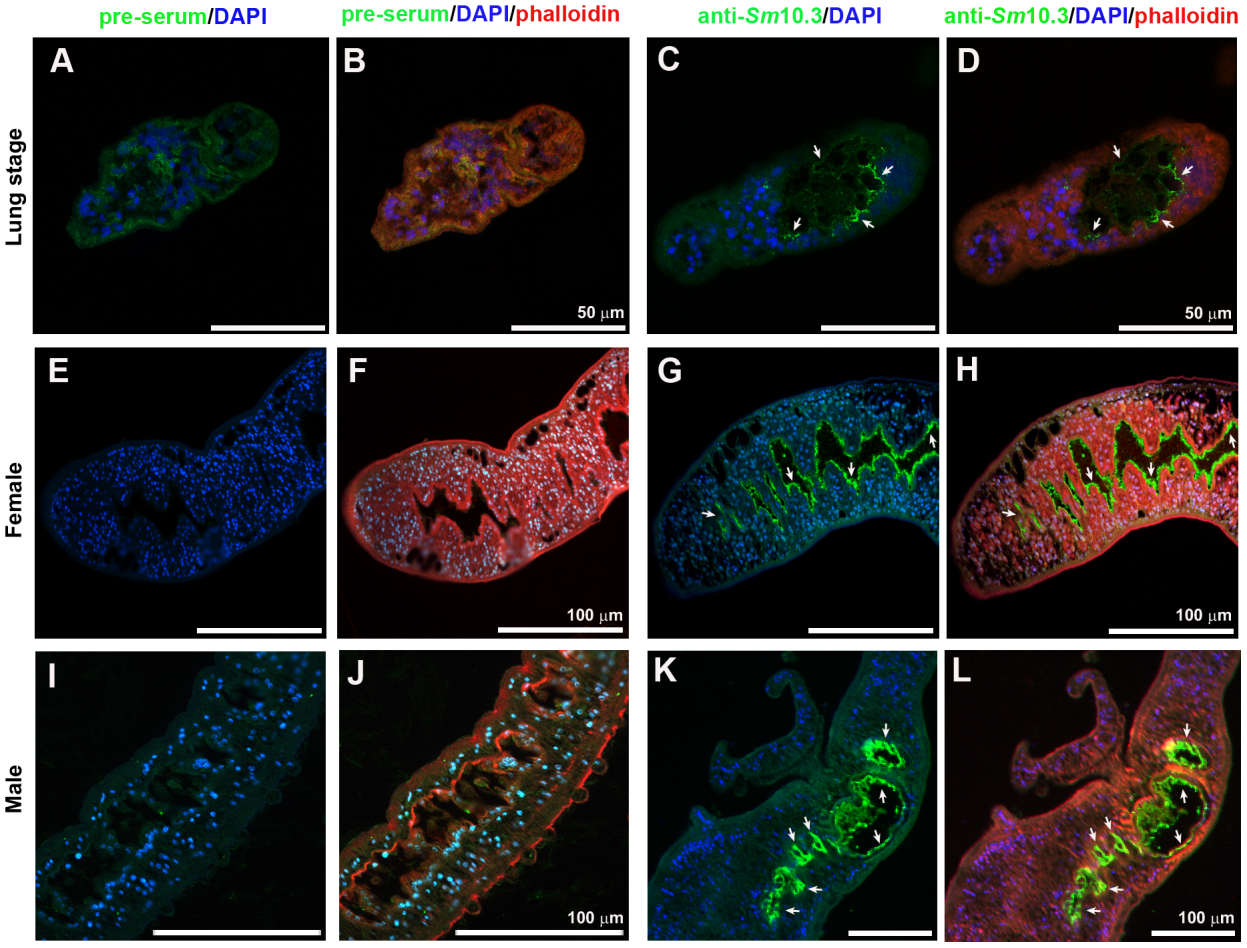
Immunolocalization of *Sm*10.3 in *S. mansoni* adult worms and lung-stage schistosomula. Mouse polyclonal anti-r*Sm*10.3 and anti-mouse secondary antibodies coupled to Alexa 488 (green) were used to label the native *Sm*10.3 protein (C, D, G, H, K, L). Serum from naive mice was used as a negative control (A, B, E, F, I, J). Nuclei were stained with DAPI (blue), and actin was stained with phalloidin conjugated to rhodamine (red). White arrows indicate the localization of *Sm*10.3 in the internal tissues of the schistosomula and on the surface and lumen of the esophageal and intestinal tegument of adult worms. Schistosomula are shown as maximum Z projections of 3 planes imaged with 1.0 µm distance intervals and adult parasites are single scans.

The localization of *Sm*10.3 was determined in *S. mansoni* lung-stage schistosomula (Fig. 3C and D), female adult parasites (Fig. 3G and H) and male adult parasites (Fig. 3K and L) using specific mouse polyclonal antibodies to r*Sm*10.3 and fluorescence microscopy. Rhodamine phalloidin (red) was used as an actin marker to label the cytoskeletal tegument components and muscle layers (Fig. 3B, D, F, H, J and L). The cell nuclei were stained with DAPI (blue) (Fig. 3). The native *Sm*10.3 protein (green) was located exclusively in the internal tissues of lung-stage schistosomula (Fig. 3C and D), as well as on the surface of the esophageal and intestinal epithelia of adult parasites (Fig. 3G and H). No *Sm*10.3-specific signal was detected in sera from naïve mice (pre-serum) in either the adult parasites (Fig. 3E, F, I and J) or the lung-stage schistosomula (Fig. 3A and B).

### r*Sm*10.3 induces agglutination of mouse erythrocytes

**Figure 4:**
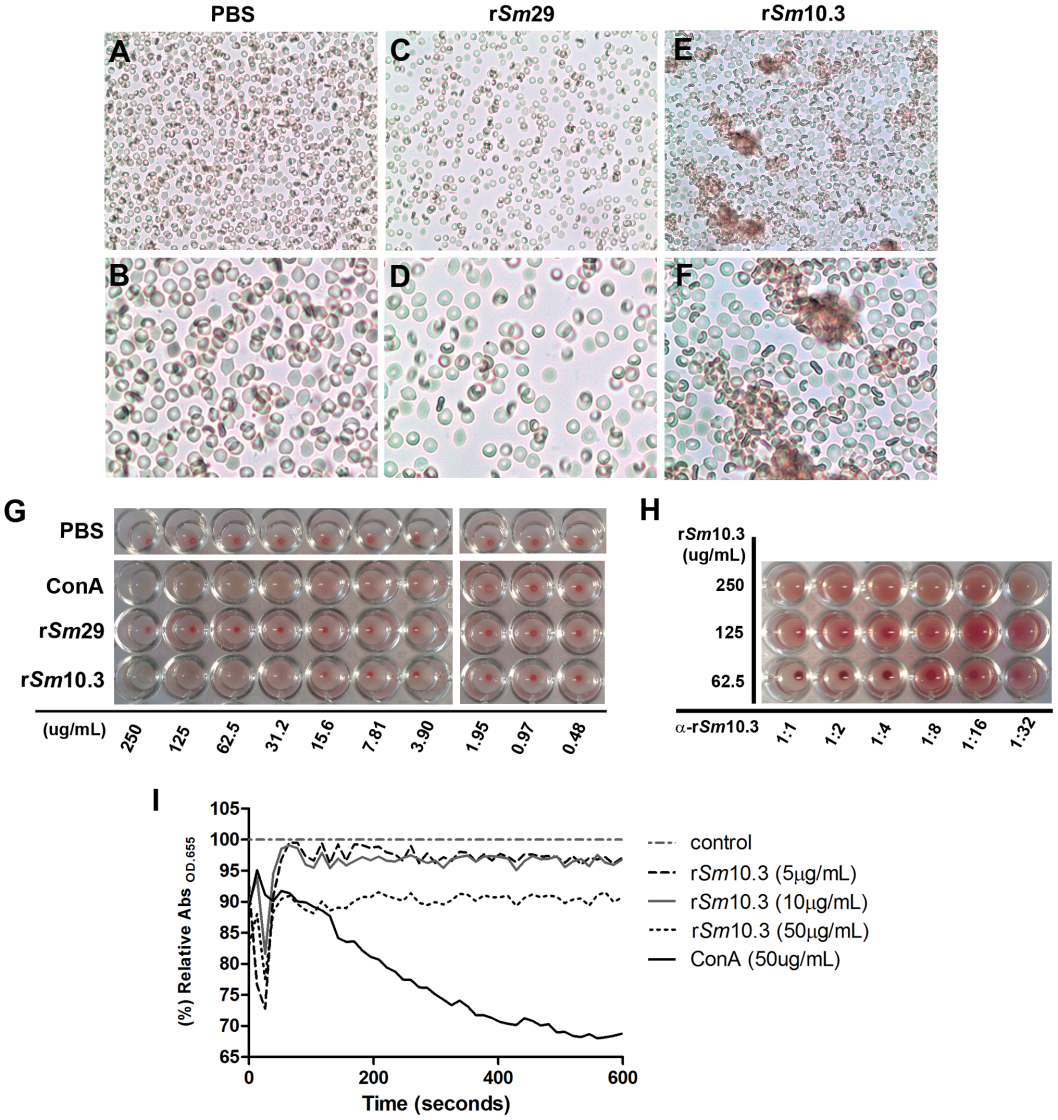
Erythrocyte agglutination assays to evaluate the hemagglutinating activity of r*Sm*10.3. *Microscopic imaging of the hemagglutinating activity of rSm10.3* (A–F). Erythrocyte suspensions (hematocrit of 20%) was combined with PBS (A, B), or 5 µg of r*Sm*29 (C, D), or 5 µg of purified r*Sm*10.3 (E, F). The mixture was loaded onto glass slides, covered with coverslips and immediately visualized at 40× (A, C, E) and 100× (B, D, F) magnification. The data shown are representative of three independent experiments. *Hemagglutination endpoint dilution assays* (G). Protein concentrations from 0.48 to 250 µg/mL of r*Sm*29, r*Sm*10.3 and concanavalin A (ConA) were tested in erythrocyte suspension (hematocrit of 1%). The deposition of erythrocytes onto the bottom of the wells, as a red dot, indicates a negative hemagglutination reaction, while the absence of erythrocytes deposition means a positive hemagglutination reaction. *Hemagglutination inhibition assays* (H). Three concentrations of r*Sm*10.3 (known to be sufficient to induce hemagglutination) were tested against six different dilutions of anti-r*Sm*10.3 antibodies, from 1∶1 to 1∶32 in a hematocrit of 1%. The deposition of erythrocytes onto the bottom of the wells indicates that the amount of anti-r*Sm*10.3 was sufficient to block r*Sm*10.3 activity, while the absence of erythrocytes deposition means that the amount of anti-r*Sm*10.3 was below the minimal concentration required to inhibit the r*Sm*10.3 hemagglutinating effect. *Kinetics of the hemagglutination process induced by rSm10.3* (I). Different concentrations of r*Sm*10.3 (5 µg/mL, 10 µg/mL and 50 µg/mL) were added to mouse erythrocytes at a final hematocrit of 2.5%. Fifty micrograms per mililiter of r*Sm*29 was used as negative control and 50 µg/mL of ConA was used as positive control. The absorbance values for r*Sm*29 were set as 100% and the absorbance readings for r*Sm*10.3 and ConA were compared to this standard. The data shown are representative of three independent experiments.

To evaluate the role of *Sm*10.3 in the digestive epithelia of adult worms and its possible contribution to the erythrocyte feeding process, we analyzed the effect of r*Sm*10.3 on mouse erythrocytes suspended in PBS. The kinetics of the hemagglutination process were monitored analyzing variations in turbidity by an adapted protocol that was previously described [26]. This methodology allows the measurement of hemagglutination due to the formation of erythrocyte clumps and a subsequent reduction in absorbance. Different amounts of r*Sm*10.3 were added to erythrocyte suspensions at a haematocrit of 5% and followed over time by reading the absorbance at 655 nm with a spectrophotometer. The recombinant protein r*Sm*29 [11] was used as a negative control and the lectin concanavalin A as a positive control of the erythrocytes hemagglutination process, as previously demonstrated [29]–[31]. The addition of a similar amount of r*Sm*29 did not induce any changes in the absorbance. However, the addition of 5 µg/mL or 10 µg/mL of r*Sm*10.3 resulted in a 5% reduction in absorbance, and higher amounts of r*Sm*10.3 (50 µg/mL) reduced the absorbance by 10–15% as compared to r*Sm*29, while 50 µg/mL of concanavalin A decreased the absorbance by 28–32% after 400 s compared to r*Sm*29 (Fig. 4I). The hemagglutination process induced by r*Sm*10.3 can also be observed microscopically, as shown by the formation of erythrocytes clumps upon the addition of recombinant *Sm*10.3 (Fig. 4E and F).

Later, the hemagglutinating effect of r*Sm*10.3 in mice erythrocytes was clearly evidenced by hemagglutination endpoint dilution assays. Protein concentrations of r*Sm*29 up to 250 µg/mL were not able to induce hemagglutination (Fig. 4G), while r*Sm*10.3 caused erythrocytes agglutination with the lowest protein concentration of 31.2 µg/mL (Fig. 4G). The minimal protein concentration of concanavalin A required to induce hemagglutination was 3.9 µg/mL, approximately eight times lower than the amount observed for r*Sm*10.3 (Fig. 4G). These results confer to concanavalin A a total hemagglutinating activity of 1.56×10^2^ units (U) and a specific hemagglutinating activity of 40000 U/mg/mL (Table S1), while these values for r*Sm*10.3 were 13 times lower and 100 times lower, respectively, 0.12×10^2^ units and 400 U/mg/mL (Table S1).

By means of hemagglutination inhibition assays using anti-r*Sm*10.3 it was demonstrated the specific and protein concentration dependent pattern of the r*Sm*10.3 hemagglutinating activity (Fig. 4H). There was no hemagglutination inhibition with 250 µg/mL of r*Sm*10.3, even at the highest anti-r*Sm*10.3 concentration (1∶1 dilution). However, with lower r*Sm*10.3 concentrations its hemagglutinating activity was inhibited by anti-r*Sm*10.3 (Fig. 4H). When using 125 µg/mL of r*Sm*10.3 there was inhibition at anti-r*Sm*10.3 dilutions ranging from 1∶1 to 1∶8, while with 62.5 µg/mL of r*Sm*10.3 the inhibition was evident from 1∶1 to 1∶16 dilutions (Fig. 4H).

### Antibody profile following the immunization of mice with r*Sm*10.3

**Figure 5:**
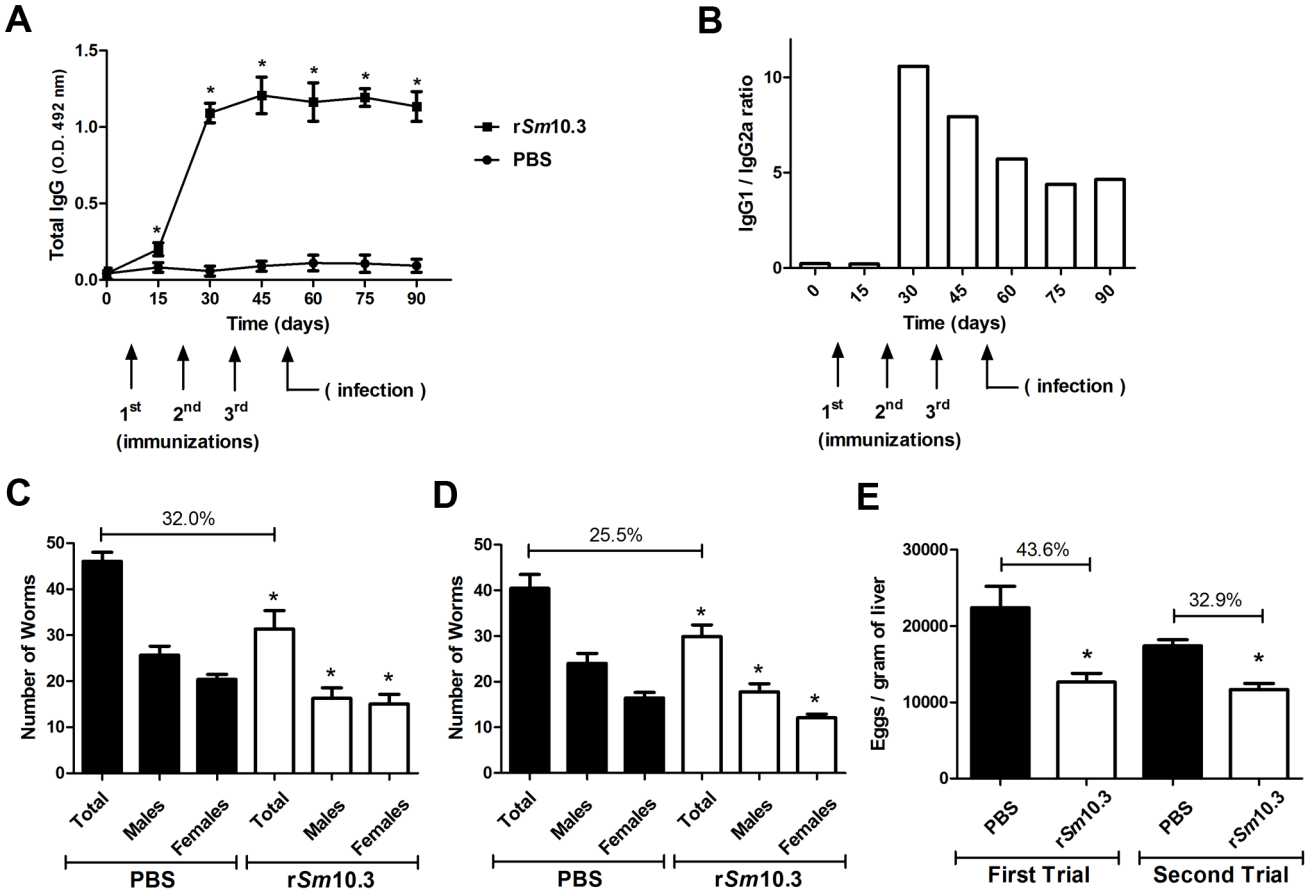
Vaccination with r*Sm*10.3 induces protective immunity in mice. (A), Sera from ten animals from each vaccination group were tested by ELISA to evaluate the levels of specific IgG antibodies to r*Sm*10.3. Significant titers of anti-r*Sm*10.3 IgG antibodies were detected at all time points tested after the first immunization. Arrows indicate when the three immunizations were administered and the infection was performed. The results shown are representative of two independent experiments. (B), IgG1 and IgG2a were tittered by ELISA as described in the material and methods and their values were used to calculate the IgG1/IgG2a ratio. Arrows indicate when the three immunizations were administered and the infection was performed. (C, D), Worm burden from two independent experiments of mice immunized with r*Sm*10.3 and challenged with live *S. mansoni* cercariae. Mice vaccinated with r*Sm*10.3 showed a 32.0% reduction in the adult worm burden in the first trial and 25.5% reduction in the second trial. (E), Oogram showing 43.6% and 32.9% reduction in the number of eggs per gram of liver tissue in the first and second trials, respectively. Error bars indicate intra-assay standard deviation of means. Asterisks indicate statistically significant differences between the vaccinated groups and the control group (*p*<0.05).

Sera from ten animals from each vaccination group were tested by ELISA to evaluate the levels of specific IgG, IgG1 and IgG2a antibodies to rSm10.3. Significant titers of anti-r*Sm*10.3 IgG antibodies were detected at all time points tested after the first immunization (Fig. 5A). The levels of specific IgG1 antibodies increased at 30 days after the first immunization (Fig. S1A), while the levels of specific IgG2a antibodies continued to increase up to day 75 (Fig. S1B). Furthermore, the IgG1/IgG2a ratio was reduced at days 45, 60, 75 and 90 (Fig. 5B), which correlates with the elevation in anti-r*Sm*10.3 IgG2a production (Fig. S1B).

### Immunization with r*Sm*10.3 induces protective immunity in mice

**Figure 6:**
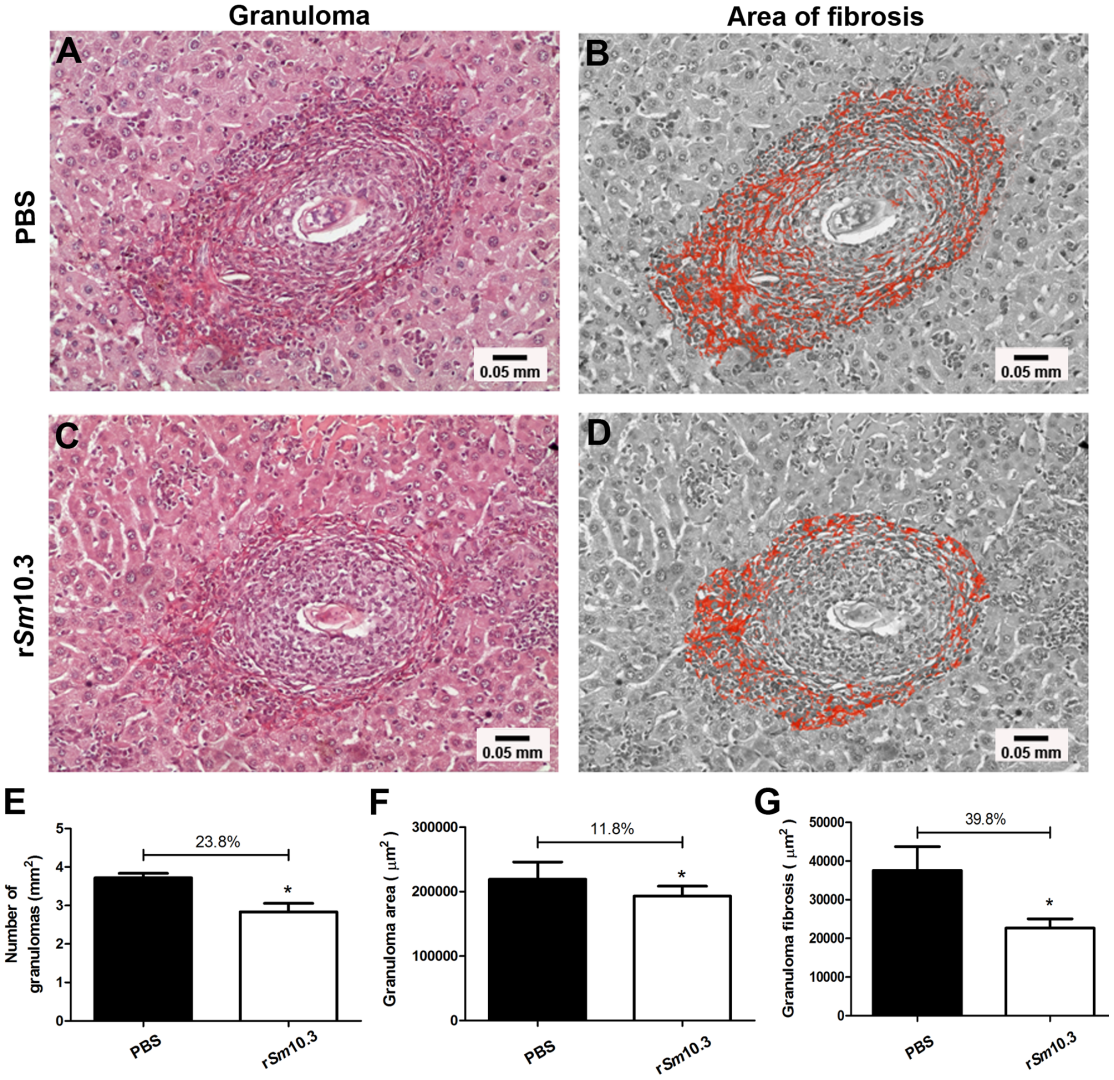
Histological analysis of hepatic tissue from mice vaccinated with r*Sm*10.3. The r*Sm*10.3-vaccinated and control animals from the first vaccination trial were sacrificed, and their livers were washed with PBS and stored in formaldehyde until sectioning and staining with picrosirius. (A) A representative sample from the PBS control group. (C) A representative sample from a mouse vaccinated with r*Sm*10.3. (B and D) Images were edited using image software to highlight the fibrotic areas in red. The images were captured using a 40× objective lens. (E), Graph showing a 23.8% reduction in the number of granulomas in vaccinated mice. (F), Graph showing a 11.8% reduction in the granuloma area in vaccinated mice. (G), Graph showing a 39.8% reduction in the granuloma fibrosis in vaccinated mice. Twenty granulomas with a single-well-defined egg were randomly selected in each liver section for the granuloma analysis. Error bars indicate intra-assay standard deviation of means. Asterisks indicate statistically significant differences between the vaccinated groups and the control group (*p*<0.05).

To test the potential usefulness of r*Sm*10.3 as part of an anti-schistosome vaccine, we asked whether this recombinant antigen could induce protection in a murine model of *S. mansoni* infection. Two independent vaccination trials were conducted and C57BL/6 mice were immunized three times with r*Sm*10.3 formulated with Freund's adjuvant and then challenged with 100 *S. mansoni* cercariae. The control group received adjuvant only in phosphate-buffered saline. Mice vaccinated with r*Sm*10.3 showed a 32.0% reduction in the adult worm burden in the first trial (Fig. 5C) and 25.5% reduction in the second trial (Fig. 5D). Regarding the number of eggs in mice livers, it was observed 43.6% and 32.9% reduction in the number of eggs per gram of liver tissue in the first and second trials, respectively (Fig. 5E).As shown in Figure 6A–D, histopathological analysis of the hepatic tissue, from animals of the first vaccination trial, demonstrated that r*Sm*10.3 immunization reduced the extent of fibrosis compared to control animals. These analysis showed a 23.8% reduction in liver granuloma counts (Fig. 6E), an 11.8% reduction in granuloma area (Fig. 6F), and a 39.8% reduction in granuloma fibrosis (Fig. 6G), as compared to control mice.

### r*Sm*10.3 immunization induces a mixed Th1/Th2 cytokine profile

**Figure 7:**
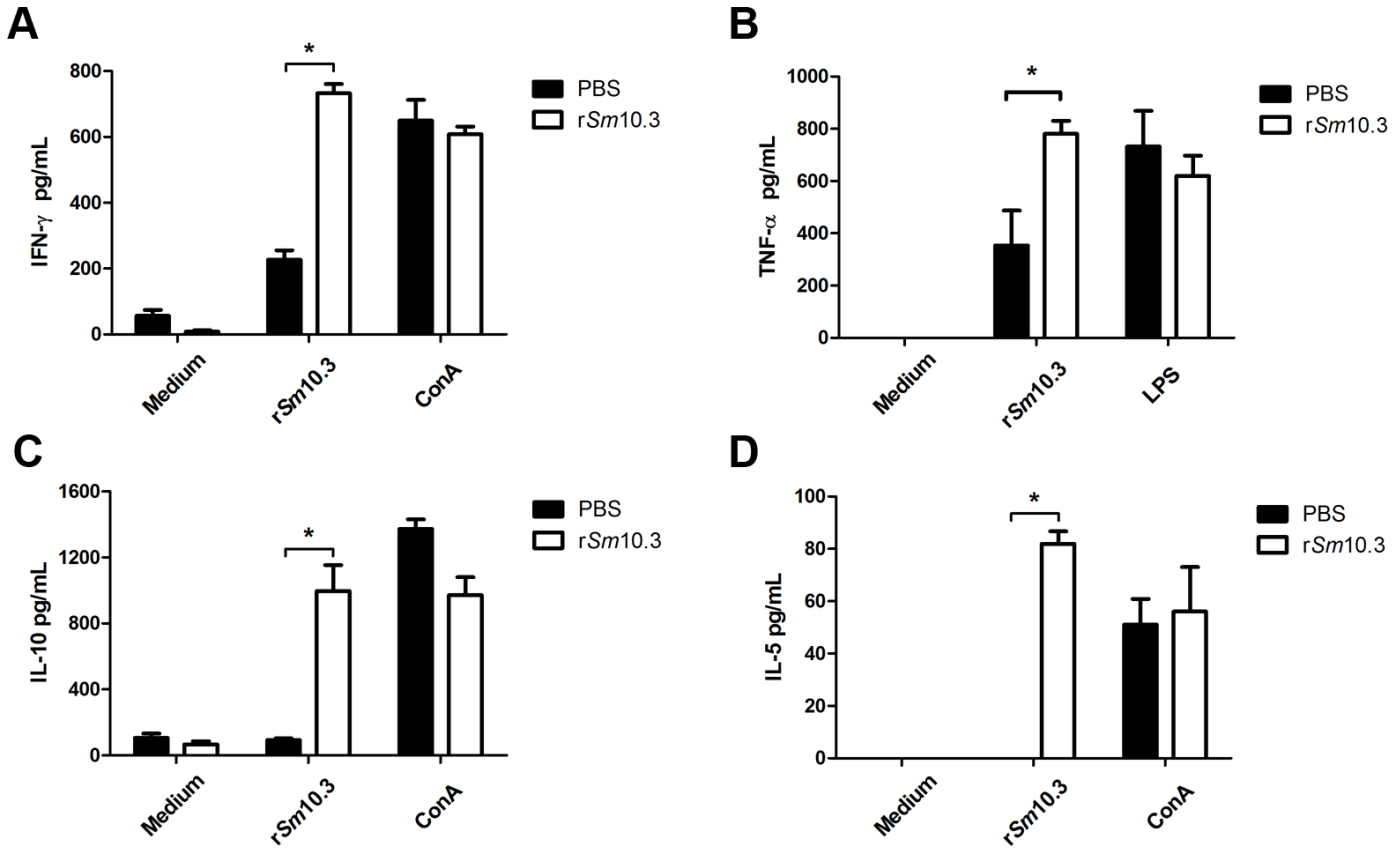
Cytokine profile of mice immunized with r*Sm*10.3. One week after the final immunization with r*Sm*10.3, splenocytes from five mice were isolated and assayed for IFN-γ (A), TNF-α (B), IL-10 (C) and IL-5 (D) production in response to medium, r*Sm*10.3 (25 µg/ml), ConA and LPS, as indicated. The control group was immunized with PBS plus Freund's adjuvant (CFA/IFA). The results are presented as the mean ± SD for each group. A statistically significant difference between the cytokines produced by splenocytes from the r*Sm*10.3-vaccinated mice and the control group is denoted by an asterisk (*p*<0.05). The results shown are representative of three independent experiments.

To evaluate the cytokine profile of mice immunized with r*Sm*10.3, splenocytes were isolated from spleens of vaccinated and control animals after the third immunization. Statistically significant levels of IFN-γ, the signature cytokine of a Th1-type immune response, and TNF-α, a pro-inflammatory cytokine, were detected in the supernatant of cultured splenocytes from immunized animals compared to the control group (Fig. 7A and B, respectively). IL-5, a characteristic Th2-type cytokine, was also detected in the supernatant of cultured splenocytes from r*Sm*10.3-immunized mice at statistically significant levels compared to the PBS control (Fig. 7-D). Furthermore, high levels of the modulatory cytokine IL-10 were also observed in vaccinated animals (Fig. 7C). Concanavalin A (ConA) and LPS were used as positive controls to confirm that the splenocytes were responsive to stimuli. As shown in Figure 7, ConA induced the production of IFN-γ, IL-5 and IL-10, while LPS induced the production of TNF-α.

## Discussion

Schistosomiasis is one of the most important neglected tropical diseases. Effective control is unlikely in the absence of improved sanitation and the development of a vaccine. Proteins located at the host/parasite interface, particularly molecules that are secreted or surface-exposed on the tegument and the intestinal epithelia, are the most promising targets for developing an anti-schistosomiasis vaccine [18]. We therefore evaluated the potential of the *Sm*10.3 antigen as a vaccine candidate, as it was previously reported to be localized on the esophageal gland in schistosomula and adult worms [15], as well as on the gut primordium of the nonfeeding cercaria [15]. The deduced amino acid sequence of *Sm10.3* contains a signal peptide, and the protein was predicted to be secreted or localized to the exterior surface of the cell. The gene products of several other MEG family members contain signal peptides for secretion and are secreted from different schistosomal glands and epithelia [20], [15].

We confirmed previous reports [20], [15] that *Sm10.3* is mainly expressed in the schistosomulum stage, as well as in other stages that involve contact with the mammalian host, such as eggs, cercariae and adult worms. MEG genes are difficult to clone, primarily due to extensive alternative splicing that generates variant transcripts of different sizes through exon skipping and the arbitrary combination of exons [19], [20], [15]. This variation in MEG gene products may represent a strategy used by members of the *Schistosoma* genus to confuse the host immune system, similar to the mechanisms of surface protein variation in *Trypanosoma brucei* and *Plasmodium falciparum* [20], [15], [18]. In this study, we produced the recombinant *Sm*10.3 protein from a synthetic gene that allowed us to express a protein corresponding to the largest transcript from the *Sm10.3* gene to optimize codon usage and avoid errors in the amino acid sequence.

Our fluorescence microscopy data confirm the *in silico* prediction that *Sm*10.3 is secreted or located on the cell surface, demonstrating that the native *Sm*10.3 protein localizes to the epithelia and lumen of the intestinal tract in adult parasites, as well as to the internal tissues of lung-stage schistosomula. A previous microarray study demonstrated an increase in *Sm10.3* expression in lung-stage schistosomula [15]. In the same study, *Sm*10.3 localization was examined using an antibody against a synthetic peptide (immunocytochemistry) and RNA hybridization (WISH). The authors found *Sm*10.3 proteins in the esophageal gland and the esophageal lumen of adult worms, but were unable to define the localization of *Sm*10.3 in the larval stage [15]. A more recent study from this group demonstrated that the *Sm*10.3 antigen is highly O-glycosylated, which means that native *Sm*10.3 a very sticky macromolecule that is highly likely to adhere to surfaces [35]. The authors suggest that this adherence could be responsible for the immunodetection of *Sm*10.3 not only in the esophageal gland but also throughout the entire esophagus (distal to secretion sites) [35], [15]. We did not detect any *Sm*10.3 accumulation in the esophageal glands, but we did find *Sm*10.3 on the esophageal epithelia, in the esophageal lumen and in the gut epithelia. It was observed immunoreactivity in the gut epithelia and sometimes in the gut lumen in all samples that were imaged. However, it was not possible to obtain image sections showing the entire gut length, which prevent us to state that *Sm*10.3 is present in the whole gut. These apparent differences in *Sm*10.3 localization could be due to the different antibodies that were used in the studies. Alternatively, variant *Sm*10.3 transcripts could be differentially regulated depending on the experimental conditions. It has been previously shown that environmental stimuli can affect gene expression in *S. mansoni*, as the presence or absence of erythrocytes altered the transcription levels of genes expressed in the tegument and related to feeding [14].

To further investigate the role of *Sm*10.3 protein in the esophagus and gut of adult worms, we analyzed the effect of r*Sm*10.3 protein on erythrocytes and found that r*Sm*10.3 induced hemagglutination *in vitro*. This effect could be related to erythrocyte digestion and nutrition in adult worms, and may represent a role for *Sm*10.3 in adult worms. However, it is necessary to evaluate *in vivo* the impact of this *Sm*10.3-induced hemagglutination on the blood feeding process. In addition to the possible role of *Sm*10.3 protein in blood feeding, it is also likely that *Sm*10.3 contributes to protein variation, a role that has been previously proposed for the MEG family members [20], [15], [18]. The digestive tracts of schistosomes in the blood feeding stages are accessible to macromolecules such as albumin and immunoglobulins [36], [37], which implies that there may be direct contact between the digestive epithelia and the host immune system.

We also assessed the interaction of *Sm*10.3 with the host immune system and its potential as a vaccine candidate. r*Sm*10.3 induced high levels of anti-r*Sm*10.3 IgG production in the sera of immunized mice after the second immunization. A decrease in the ratio between the IgG subtypes (IgG1/IgG2a) was observed 45 days after the first immunization. Furthermore, cytokine analysis of the supernatants of cultured splenocytes stimulated with r*Sm*10.3 suggests a mixed Th1/Th2-type immune response. Studies using the irradiated cercariae model, which induces high levels of protection in mice, suggest that effective protection can be based on a mixed Th1/Th2 response, a polarized Th1 response or even a polarized Th2 response [38]. In previous studies performed by our group, the majority of *S. mansoni* antigens tested as recombinant protein vaccines that conferred partial protection against cercariae challenges induced a Th1-type immune response [11], [34], [39], [40] or a mixed Th1/Th2 response [41], [42], [43]. IFN-γ is involved in protective immunity against schistosomiasis, as specific anti-IFN-γ antibodies completely abolish the protection conferred by vaccination with irradiated cercariae [44]. Similar results were obtained in a study using IFN-γ knockout mice [45]. The partial protection conferred by vaccination with r*Sm*10.3 resulted in 25.5% to 32% reduction in worm burden, and the overall pathology was reduced, as shown by 32.9% to 43.6% reduction in the number of eggs per gram of hepatic tissue, a 23.8% reduction in the number of granulomas, an 11.8% reduction in the area of the granulomas and a 39.8% reduction in granuloma fibrosis. It is possible that the reduced liver pathology is related to the elevated levels of IL-10 detected in immunized mice, which may regulate Th2 responses and/or prevent the development of a polarized Th1 response, consequently reducing inflammation and liver injury [46], [47].

In conclusion, our results confirm that *Sm*10.3 is mainly expressed during the stages of the *Schistosoma mansoni* life cycle that involve contact with the mammalian host. We show that the *Sm*10.3 protein is located in the intestinal tract of adult worms, providing the first evidence for a possible role for *Sm*10.3 in the blood feeding process. Finally, our data suggest that *Sm*10.3 is a potential candidate for use in developing a multi-antigen vaccine to control schistosomiasis.

## Supporting Information

Figure S1 **Kinetics of specific IgG1 and IgG2a anti-r** ***Sm*** **10.3 production in the sera of vaccinated mice.** Sera from 10 immunized mice per group were collected prior to the first immunization and at days 15, 30, 45, 60, 75 and 90 after the first immunization. The control group was injected with PBS plus Freund's adjuvant. The sera were assayed by ELISA for specific IgG1 and IgG2a antibodies. Arrows indicate when the three immunizations were administered. The results are presented as the mean absorbance at 492 nm for each group. Asterisks indicate statistically significant differences between the vaccinated groups and the control group (*p*<0.05). Error bars indicate intra-assay standard deviation of means. The results shown are representative of two independent experiments.
(TIF)

Table S1 **Hemagglutinating activity of r** ***Sm*** **10.3 in mouse erythrocytes.** The results were read after approximately 1 h when the blank had fully sedimented (See Fig. 4G). The endpoint was defined as the highest dilution showing complete hemagglutination. The hemagglutination titer, defined as the reciprocal of the highest dilution exhibiting hemagglutination, was defined as one hemagglutination unit. Specific activity is the number of hemagglutination units per mg of protein per milliliter.
(PDF)
