# Supplementary figures and images for: Sm10.3, a Member of the Micro-Exon Gene 4 (MEG-4) Family, Induces Erythrocyte Agglutination In Vitro and Partially Protects Vaccinated Mice against Schistosoma mansoni Infection
Source: PLoS Negl Trop Dis. 2014 Mar 20;8(3):e2750. doi: 10.1371/journal.pntd.0002750 (PMC3961193; doi:10.1371/journal.pntd.0002750)

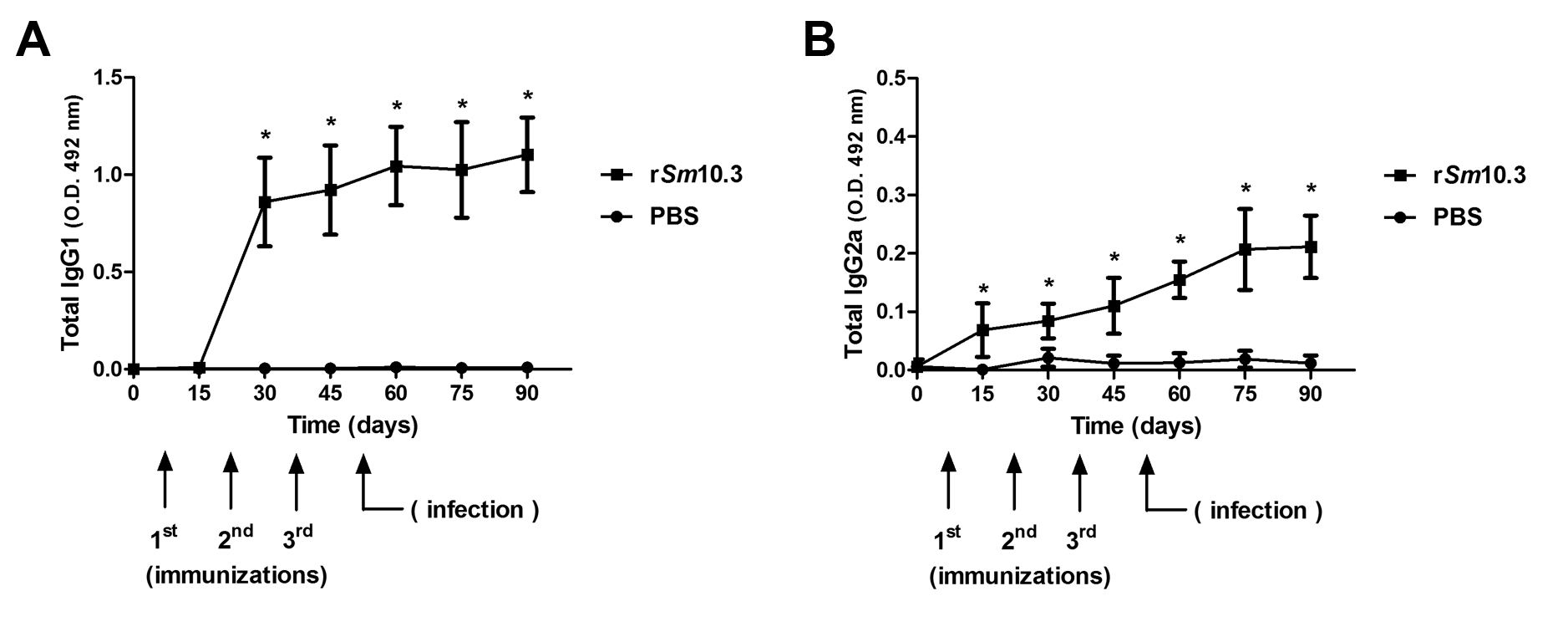

Supplement: Figure S1 — Kinetics of specific IgG1 and IgG2a anti-r Sm 10.3 production in the sera of vaccinated mice. Sera from 10 immunized mice per group were collected prior to the first immunization and at days 15, 30, 45, 60, 75 and 90 after the first immunization. The control group was injected with PBS plus Freund's adjuvant. The sera were assayed by ELISA for specific IgG1 and IgG2a antibodies. Arrows indicate when the three immunizations were administered. The results are presented as the mean absorbance at 492 nm for each group. Asterisks indicate statistically significant differences between the vaccinated groups and the control group (p<0.05). Error bars indicate intra-assay standard deviation of means. The results shown are representative of two independent experiments. (TIF) [file pntd.0002750.s001.tif]
