# Supplementary material for: Sm10.3, a Member of the Micro-Exon Gene 4 (MEG-4) Family, Induces Erythrocyte Agglutination In Vitro and Partially Protects Vaccinated Mice against Schistosoma mansoni Infection
Source: PLoS Negl Trop Dis. 2014 Mar 20;8(3):e2750. doi: 10.1371/journal.pntd.0002750 (PMC3961193; doi:10.1371/journal.pntd.0002750)
Supplement: Table S1 — Hemagglutinating activity of r Sm 10.3 in mouse erythrocytes. The results were read after approximately 1 h when the blank had fully sedimented (See Fig. 4G). The endpoint was defined as the highest dilution showing complete hemagglutination. The hemagglutination titer, defined as the reciprocal of the highest dilution exhibiting hemagglutination, was defined as one hemagglutination unit. Specific activity is the number of hemagglutination units per mg of protein per milliliter. (PDF) [file pntd.0002750.s002.pdf]

Table S1. Hemagglutinating activity of r*Sm*10.3 in mouse erythrocytes

Proteins	Total protein (mg/mL)	Total hemagglutinating activity (U)	Specific hemagglutinating activity (U/mg/mL)
**ConA**	3.9×10^{-3}	1.56×10^{2}	40000
**r*Sm*10.3**	31.2×10^{-3}	0.12×10^{2}	400
